# Maintenance of Yeast Genome Integrity by RecQ Family DNA Helicases

**DOI:** 10.3390/genes11020205

**Published:** 2020-02-18

**Authors:** Sonia Vidushi Gupta, Kristina Hildegard Schmidt

**Affiliations:** 1 Department of Cell Biology, Microbiology and Molecular Biology, University of South, Florida, Tampa, FL 33620, USA; soniavidushi@mail.usf.edu; 2 Cancer Biology and Evolution Program, H. Lee Moffitt Cancer Center and Research, Institute, Tampa, FL 33612, USA

**Keywords:** RecQ helicases, Sgs1, Rqh1, yeast, genome instability

## Abstract

With roles in DNA repair, recombination, replication and transcription, members of the RecQ DNA helicase family maintain genome integrity from bacteria to mammals. Mutations in human RecQ helicases BLM, WRN and RecQL4 cause incurable disorders characterized by genome instability, increased cancer predisposition and premature adult-onset aging. Yeast cells lacking the RecQ helicase Sgs1 share many of the cellular defects of human cells lacking BLM, including hypersensitivity to DNA damaging agents and replication stress, shortened lifespan, genome instability and mitotic hyper-recombination, making them invaluable model systems for elucidating eukaryotic RecQ helicase function. Yeast and human RecQ helicases have common DNA substrates and domain structures and share similar physical interaction partners. Here, we review the major cellular functions of the yeast RecQ helicases Sgs1 of *Saccharomyces cerevisiae* and Rqh1 of *Schizosaccharomyces pombe* and provide an outlook on some of the outstanding questions in the field.

## 1. The RecQ Helicase Family Is Conserved from Bacteria to Humans

To maintain genome integrity, RecQ-like DNA helicases act as key factors in homologous recombination (HR) and recombinational DNA repair and, in some organisms, perform accessory roles in DNA replication and transcription. The RecQ helicase family owes its name to its discovery in *Escherichia coli* where a new mutation, *recQ1*, was identified while screening for mutations conferring resistance to thymineless death [1,2]. Thymineless death is the phenomenon of cell death in prokaryotes and eukaryotes that occurs upon depletion of the DNA base thymine. The exact mechanism is still under investigation, but it requires a RecQ-dependent pathway of homologous recombination that also includes RecJ, a RecA-dependent pathway that involves the SOS-response, and possibly a yet unidentified third pathway [3,4,5]. Disruption of RecQ significantly reduces recombination frequency and increases sensitivity to ultraviolet (UV) radiation of cells in which recombination events are prevented from proceeding through RecBCD and SbcBC pathways, identifying RecQ as a member of the alternate RecF recombination pathway [1,6]. In wildtype cells, this RecF pathway appears to mediate genetic exchange at single-strand (ss) DNA gaps and contributes to the repair of collapsed replication forks. Since then, RecQ helicases have been identified in all organisms, from bacteria to plants and humans. Unicellular organisms typically express a single RecQ-like DNA helicase, such as RecQ in *E. coli*, Sgs1 in *S. cerevisiae* and Rqh1 in *S. pombe*, although evidence of additional RecQ helicases in yeasts has recently emerged [7]. In contrast, multiple RecQ-like helicases are present in multicellular eukaryotes, with seven in *Arabidopsis thaliana* currently holding the top spot [8]. The human genome encodes five RecQ-like DNA helicases (RecQL1 to RecQL5), three of which are associated with genetic disorders. Inactivation of RecQL2 (WRN), RecQL3 (BLM), and RecQL4 leads to Werner’s syndrome, Bloom syndrome and Rothmund–Thomson syndrome, respectively; these disorders are characterized by genome instability, increased cancer risk and, in the case of Werner syndrome, adult-onset premature aging [9,10,11,12]. Additional symptoms of Bloom syndrome include short stature and increased risk for Type-2 diabetes, immunodeficiency, infertility, and sun-sensitivity [13].

Based on mutant phenotypes, protein structure and protein–protein interactions, *S. cerevisiae* Sgs1 is most closely related to human BLM [9,14]. Loss of Sgs1 results in hypersensitivity to DNA damaging agents such as methylmethanesulfonate (MMS) and hydroxyurea (HU) [15,16], increased gross chromosomal rearrangements (GCRs) [17], reduced replicative lifespan [18], increased rate of mitotic recombination [19] and frequent chromosome missegregation [20], highlighting the importance of Sgs1 for maintaining yeast genome integrity. Similarly, *rqh1* mutants of *S. pombe* are hypersensitive to HU and UV, display chromosome missegregation, elevated recombination and are defective in recovery from S phase arrest [21,22,23]. This review highlights the ever-expanding cellular functions of RecQ helicases in yeast.

## 2. Domain Structure of the Sgs1 and Rqh1 Helicases

**Figure 1:**
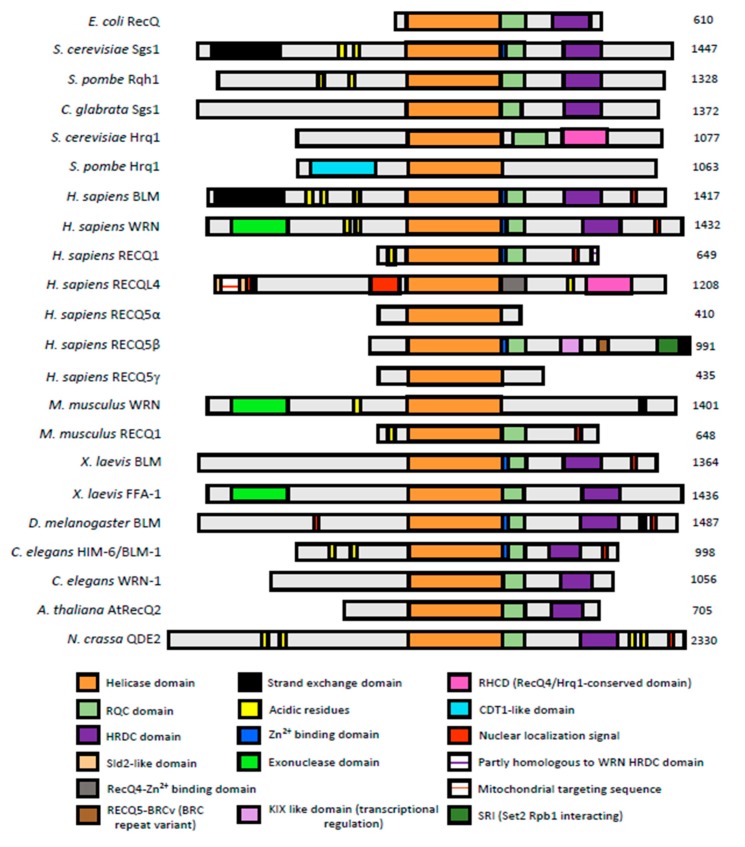
**Conserved domain structure of RecQ helicases from major model systems.** RecQ helicases are conserved from bacteria to mammals. Proteins are aligned by their conserved helicase domains. The respective organism is shown on the left and the protein length in amino acids is indicated on the right. Human RECQ5α, RECQ5β and RECQ5γ are isoforms resulting from alternative splicing of the *RECQ5* gene.

The RecQ helicase family belongs to the superfamily 2 (SF2) helicases. They are motor proteins with 3′-5′ DNA helicase activity and unwind DNA in an ATP-dependent manner, requiring Mg^2+^ as a cofactor [24]. All RecQ-like DNA helicases possess a helicase domain of nearly 400 amino acids (Figure 1) containing the seven conserved ATPase/helicase motifs (I, Ia, II, III, IV, V, VI) and a characteristic ‘motif 0′ upstream of motif I [21,25,26,27]. Motif 0 is involved in ATP binding in a manner that is similar to that of the Q motif of DEAD-box helicases, which has led to the suggestion of a possible evolutionary connection between the DNA and RNA helicases of the RecQ and DEAD-box families, respectively [28,29]. These conserved motifs form the surface of the catalytic cleft between the two RecA-like lobes where ATP is hydrolyzed in a ssDNA-dependent manner [30,31]. Human BLM possesses a unique proline/lysine-rich loop that protrudes from the surface of the second RecA-like lobe. Although its precise function is unknown, its functional significance is supported by a mutation that partially inactivates BLM, causing increased sister-chromatid exchanges and a slow double-strand break repair phenotype [32,33]. With an allele frequency of 5.3%, this mutation (P868L) is not associated with Bloom syndrome, but may be a candidate for a new cancer risk allele in otherwise healthy individuals [33].

Helicase-and-RNaseD-like-C-terminal (HRDC) and RecQ C-terminal (RQC) domains are present in bacterial RecQ, including RecQ helicases with multiple HRDC domains [34], the yeast RecQ helicases Sgs1 and Rqh1, and in most RecQ helicases of multicellular organisms (Figure 1). The RQC domain consists of a winged-helix (WH) domain and a zinc-binding domain; the latter has been implicated in structural stability of the protein [35,36] whereas the WH domain acts as a DNA binding motif in many proteins [37,38,39,40]. Structural and biochemical analyses for *E. coli* RecQ and WRN indicate that the WH domain can interact with dsDNA [30,41,42]. The HRDC domain is dispensable for ATPase activity and unwinding of simple double-stranded DNA substrates, but contributes to DNA binding and DNA substrate specificity which, for example, is required for double-Holliday-junction dissolution by BLM [43]. Other structural analyses of BLM and Sgs1 have detected low or no DNA-binding affinity of the HRDC domain, but identified an intramolecular interaction between the BLM HRDC domain and the ATPase domain that may affect helicase activity [31,44]. The HRDC domain in RecQ helicases has not been directly implicated in mediating protein–protein interactions, but it is part of a fragment of human BLM that binds the telomere-associated protein Trf2 [45].

**Figure 2:**
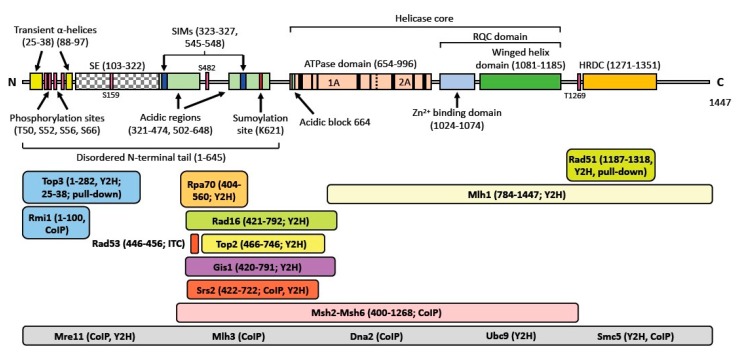
**Structure of Sgs1 and its binding partners.** Sgs1 is composed of the structurally ordered ATPase/helicase core and HRDC domains in the C-terminal half of the protein and a 645-residue long intrinsically disordered N-terminal tail. This domain structure is conserved in human BLM and several other RecQ family helicases. In addition to SUMOylation at K621, SUMOylation at K175 and K831 has also been reported [54]. Pink boxes indicate phosphorylation sites. The helicase core contains the ATPase domain, consisting of two RecA-like lobes, 1A and 2A, which harbor the eight RecQ conserved helicase motifs indicated in black (motifs 0 to VI), and the RQC-domain, consisting of the zinc-binding and winged-helix subdomains. For interacting proteins, the binding region in Sgs1 is shown in brackets after the protein name, followed by the assay of detection. Interacting proteins for which binding sites on Sgs1 have not been narrowed down are listed in the gray box. SE, strand-exchange domain; SIMs, SUMO-interacting motifs; RQC, RecQ-C-terminal domain; HRDC, helicase-and-RNaseD-like-C-terminal domain; Y2H, yeast-two-hybrid assay; co-IP, co-immunoprecipitation; ITC, isothermal titration calorimetry.

In addition to conserved, structured domains that make up the central helicase core comprised of the ATPase and RQC domains, most eukaryotic RecQ helicases possess long, disordered N-terminal tails. With the exception of WRN, whose N-terminal tail harbors a 3′-5′ exonuclease domain [46,47,48], these disordered tails are devoid of catalytic activity and act as binding sites for the often-large number of interacting proteins (Figure 2). The N-terminus of Sgs1 interacts with at least six proteins, including type I topoisomerase Top3 and Rmi1, forming the STR complex for double Holliday junction dissolution, replication protein A (Rpa) 70, antirecombinase Srs2, global nucleotide excision repair factor Rad16, and the type II topoisomerase Top2. Additional protein interactions for which binding sites on Sgs1 have not yet been narrowed down to specific domains include the DNA double-strand-break (DSB) recognition factor Mre11, the single-strand specific nuclease Dna2, mismatch repair proteins Mlh1/3 and Msh2/6, the SUMO-conjugating enzyme Ubc9 and Smc5 of the Smc5/6 complex, which is a key factor for recombinational DNA repair [49,50,51,52,53,54,55,56]. Although the vast majority of protein interactions occur with the N-terminal tail of Sgs1, the interaction of Sgs1 with the strand exchange protein Rad51 occurs immediately downstream of the RQC domain, with Phe 1192 playing a crucial role in promoting homologous recombination [57].

Sgs1 possesses additional specialized motifs, of which some are conserved in BLM but not in other eukaryotic RecQ helicases. Acidic regions in the disordered N-terminal tail of Sgs1 mediate an interaction with Rpa70 [58]. RPA prevents reannealing of ssDNA and stimulates Sgs1 activity *in vitro*, and this functional relationship is conserved in human BLM [59,60,61]. It has also been proposed that the acidic regions contribute to the role of Sgs1 in promoting homologous recombination by acting as a DNA mimic that competes with ssDNA for RPA binding, thereby facilitating the initial loading of Sgs1-bound Rad51 onto 3′ ssDNA overhangs of resected DSBs and promoting Rad51 filament initiation and HR [57]. Such a role of the Sgs1/Dna2 resection machinery would be reminiscent of the role of RecBCD in promoting RecA loading in *E. coli* [62]. Finally, residues 103-322 of Sgs1 display ssDNA binding as well as strand annealing and exchange activities that enable Sgs1 to anneal complementary single DNA strands *in vitro* [7,63,64]. This activity is conserved in human BLM [65].

## 3. Post-Translational Modification of Sgs1 and Rqh1

**Figure 3:**
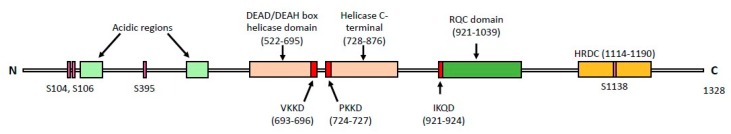
**Structure of Rqh1.** Rqh1 shares the ATPase/helicase, RQC and HRDC domains with Sgs1 and other RecQ helicases [78]. Red boxes indicate predicted SUMOylation sites with residues 724-727 (PKKD) being the predominant site. Pink boxes represent phosphorylation sites [76,77].

Post-translational modifications regulate interactions with other proteins, cellular localization of proteins, and functional activation [66]. Compared to its human counterparts, Sgs1 and Rqh1 undergo relatively modest post-translational modification. Several [S/T]Q sites in the acidic region between residues 400-600 of Sgs1 are phosphorylated in a Mec1-dependent manner during exponential growth, with T451 phosphorylation being important for Rad53 binding *in vitro*. Sgs1 was also shown to be phosphorylated at positions S256 and S259 in a Rad53-independent manner under MMS-induced replication stress [67]; however, the functional significance of this event is currently unknown. Several other phosphorylation sites whose effects on Sgs1 function are poorly understood are indicated in Figure 2 [58,68,69,70,71,72]. SUMOylation of lysine 621 is required for the role of Sgs1 in telomere-telomere recombination [70,73,74]. Similarly, SUMOylation of Rqh1 stimulates its activity at telomeres, but also promotes breakage and hyper-recombination of damaged telomeres [75]. Quantitative phosphoproteomics has identified several Rqh1 phosphorylation sites that are currently of unknown functional significance, such as S104, S106, S395, and S1138 [76,77]. Figure 3 presents the domain structure and post-translational modifications of Rqh1.

## 4. Substrate Preferences of Sgs1 and Rqh1

**Figure 4:**
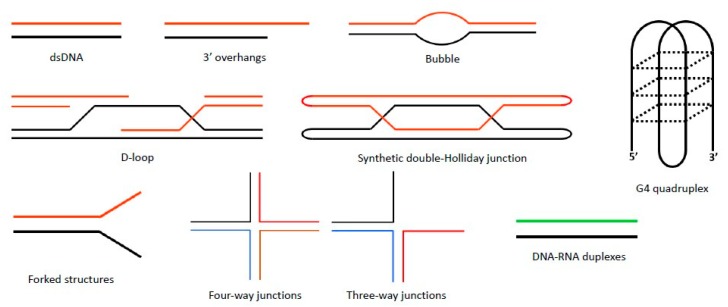
**DNA substrates that Sgs1 binds and unwinds.** Sgs1 preferentially unwinds Holliday junctions *in vitro* and G4 quadruplex DNA with more efficiency than duplex DNA. It can displace the D-loop formed during strand invasion and also unwind forked and ssDNA overhang structures.

Sgs1 acts on a wide range of DNA substrates (Figure 4). Sgs1 exhibits affinity for single and double-stranded DNA; however, its affinity for forked structures is markedly higher [73]. Notably, Sgs1 unwinds DNA-RNA duplexes with the same efficiency as DNA-DNA duplexes—an uncommon property for DNA helicases [73]. Sgs1 is able to discriminate between 3′ and 5′ overhangs and unwinds three way junctions and Holliday-junction-like four-way junctions—a key intermediate of homologous recombination [79]. Setting it apart from most RecQ helicases, Sgs1 is able to unwind G-G paired DNA, such as in G-quadruplex (G4) structures, more efficiently than duplex DNA. G4 structures can form in G-rich DNA sequences, which are particularly found in rDNA and telomeric repeats, thereby implicating Sgs1 as a protector of rDNA stability and telomeres [80,81]. The RQC domain of human BLM is a conserved G4-DNA binding domain, but such a determination remains to be made for Sgs1 or Rqh1 [82].

## 5. Physical Interactions of Sgs1 and Rqh1

Sgs1 interacts with several proteins involved in DNA recombination and the replication stress response (Figure 2). Mutations in *SGS1* rescue the severe fitness defect and genomic instability phenotypes of *top3* mutants [19]. Similarly, disruption of the *rqh1* gene in *S. pombe* rescues the inviability of *top3* mutants [83]. The first evidence of a physical interaction between Sgs1 and Top3 came from a yeast two-hybrid screen, followed by additional studies that mapped the Top3 binding domain to the N-terminal 282 amino acid residues of Sgs1 [19,70,84,85]. Similarly, the Top3-interacting domain in Rqh1 lies within the first 322 residues [78]. Along with Rmi1, Sgs1/Rqh1 and Top3 function as a single heteromeric complex. Rmi1 and Top3 interact with each other and can only bind to Sgs1 as a complex [86,87]. By binding to the topoisomerase gate, Rmi1 is thought to enhance the decatenase activity of Top3 [88,89]. Nuclear magnetic resonance spectroscopy showed that the first 125 N-terminal residues of Sgs1 are intrinsically disordered and contain two transient α-helices comprised of residues 25-38 and residues 88-97 [87]. Disrupting the first α-helix with single proline mutations (e.g., sgs1-F30P) impairs Sgs1 binding to Top3-Rmi1 and results in hypersensitivity to DNA damaging agents and accumulation of gross chromosomal rearrangements [87].

The Rad51 binding domain of Sgs1 was initially mapped to the region C-terminal of the helicase core and one critical region later narrowed down to residues 1187-1207 [57,90]. Mutation of F1192 to asparagine was sufficient to disrupt the Sgs1-Rad51 interaction *in vitro* [57]. Distinct positive and negative genetic interactions of this *sgs1-FD* allele with mutations in HR genes, such as *mre11Δ*, *sae2Δ*, *top3Δ*, *exo1Δ* and *srs2Δ*, revealed that the Sgs1-Rad51 interaction promotes HR, possibly by facilitating Rad51 filament initiation [57].

An RPA-binding domain has been mapped to amino acid residues 404-560 of Sgs1 just upstream of the helicase domain, overlapping an acidic region and the Rad53-interacting domain between residues 446-456 [58]. Mec1-mediated phosphorylation of [S/T]Q residues in this acidic region promotes Sgs1 binding to Rad53, thereby recruiting the kinase to stalled forks and promoting the replication checkpoint response [58].

Co-immunoprecipitation experiments have also identified a physical interaction of Sgs1 with the Dna2 nuclease, which functions with Sgs1 and the single-strand binding protein RPA in the long-range resection of DSBs [50]. During DSB repair, Sgs1 also interacts with the antirecombinase Srs2 at a region that spans residues 422-722 and with the Mre11 subunit of the DSB recognition complex MRX [91]. All three proteins—Sgs1, Srs2 and Mre11—form a large complex under normal as well as DNA-damaging conditions [49], which likely functions in the recruitment of these HR proteins to DSBs.

The molecular basis and functional significance of physical interactions of Sgs1 with several other proteins is less well-understood. Rad16, a protein involved in lesion recognition during global nucleotide excision repair (NER), physically interacts with the 421–792 amino acid region of Sgs1, possibly implicating Sgs1 in DNA damage recognition [92]. The Rad16-binding region overlaps with the Top2-interacting region, which spans residues 466-746. Top2 is a type-II topoisomerase and a mitotic post-replication decatenase that acts in the same pathway as Sgs1 to prevent chromosome missegregation [20,85,93]. Sgs1 also physically interacts with the mismatch-repair factors Msh2/6 and Mlh1 and this interaction is conserved in human BLM; however, the biological significance of this interaction in yeast and human cells remains to be elucidated [51,56,94,95,96]. That BLM-deficient cells are not defective in DNA mismatch repair and that in late prophase of meiosis I Sgs1 interacts with Mlh3, which forms a heteroduplex with Mlh1 and promotes meiotic crossing-over, may suggest that the interaction of Sgs1/BLM with mismatch repair proteins functions in genetic recombination rather than in DNA replication-coupled mismatch repair [52]. In a yeast two-hybrid assay, Sgs1 residues 420-791 were found to interact with the jumonji domain of Gis1, a transcription factor that has two jumonji subdomains, one of which causes histone demethylation, thereby facilitating transcription [97]. However, whether a link between DNA recombination and histone demethylation exists in yeast still remains to be seen. Using two-hybrid assays, interactions of Sgs1 with Ubc9 and SUMO were also identified, raising the possibility that Sgs1 may also be SUMOylated by Ubc9 [55]. Table 1; Table 2 summarize binding partners of the yeast RecQ helicases Sgs1 and Rqh1.

## 6. Homologous Recombination-Mediated DNA Double-Strand Break Repair

### 6.1. Genetic Interactions of SGS1 and RQH1 with HR Genes

DSBs can be repaired by either nonhomologous end joining (NHEJ), which directly ligates the broken ends and is preferred during G1 phase of the cell cycle, or homologous recombination (HR), which utilizes a sister chromatid or a homologous chromosome for restoring genetic information during the S/G2/M phases or meiosis [113]. Studies that first highlighted the roles of Sgs1 and Rqh1 in HR showed that *sgs1Δ* and *rqh1Δ* mutants exhibit mitotic hyperrecombination phenotypes [19,23,114]. For DNA-damage sensitivity and growth, homologous recombination genes *RAD55, RAD57, RAD54* and *RAD51* are epistatic to *SGS1* whereas *RAD52* is mostly independent of *SGS1* [90,115]. However, a genetic background where *SGS1* was in a different pathway for the repair of HU-induced DNA damage than *RAD51* and *RAD54* has also been reported [116]. Accumulation of gross-chromosomal rearrangements, while moderate in the *sgs1Δ* mutant, are reduced by *RAD51* deletion to the rate of the *rad51Δ* mutant, in support of the function of *RAD51* and *SGS1* in the same pathway [57].

Deletion of *SGS1* suppresses the slow-growth and hyper-recombination phenotypes of the *top3Δ* mutant, revealing that *sgs1Δ* is epistatic to *top3Δ* and suggesting that Top3 resolves a deleterious substrate produced by Sgs1 [19]. Relevant to this model is Pif1, a DNA helicase with opposite polarity of Sgs1 that functions in both nuclear and mitochondrial DNA metabolism [117]. That Pif1 overexpression suppresses *top3Δ* defects but aggravates *sgs1Δ* and *sgs1Δ top3Δ* defects, and that Pif1 localizes to S-phase specific DNA repair foci, suggests that Pif1 may prevent or repair deleterious recombination intermediates that are presumably formed when Sgs1 activity becomes uncoupled from Top3 [118]. Although an *SGS1* deletion suppresses the slow growth of the *top3Δ* mutant, it produces a growth defect in the *top1Δ* mutant [19,119], which has led to the suggestion that Sgs1-Top3 competes with another pathway involving Top1 and Srs2 for regulating initial recombination events [85]. Mutations in HR repair genes *RAD51, RAD55* and *RAD57* in *S. cerevisiae* and *rhp51* and *rhp57* in *S. pombe* suppress the synthetic lethality and severe slow growth phenotypes of *sgs1Δ srs2Δ* and *Δrqh1 Δsrs2* mutants, respectively, providing further evidence for the involvement of Sgs1 and Rqh1 in later steps of homologous recombination [120,121].

*SGS1* strongly interacts with *EXO1*. The *sgs1Δ exo1Δ* mutant exhibits a pronounced fitness defect and accumulates gross-chromosomal rearrangements at one of the highest rates observed to date, exceeding that of the single mutants by 200-fold [122,123,124]. The presence of DSBs in the *sgs1Δ exo1Δ* mutant that are only minimally trimmed by the MRX-Sae2 complex indicates that the molecular basis for the strongly negative genetic interaction between *SGS1* and *EXO1* is the disruption of long-range resection of DSBs, which provided the original evidence for redundant roles of Sgs1 and Exo1 in long-range DSB resection [122,125,126]. The dramatic decrease in viability of the *sgs1Δ exo1Δ* mutant upon deletion of HR genes *RAD59* or *RAD52,* but not *RAD51*, indicates that a Rad59/Rad52-mediated HR pathway is needed for proper repair of the incompletely processed DSBs in the *sgs1Δ exo1Δ* mutant [57].

The identification of a role for Sgs1 in homeologous recombination prompted an investigation into genetic interactions of Sgs1 with mismatch repair (MMR) genes, which regulate recombination between nonidentical (homeologous) DNA sequences [17,127]. Indeed, the *sgs1Δ msh2Δ* double mutant displayed significantly higher rates of gross-chromosomal rearrangements and recombination between homeologous sequences than either single mutant [17,127]. However, while Sgs1 suppressed recombination events between homeologous regions in the divergent genes *CAN1*, *LYP1* and *ALP1,* the mismatch repair factor Msh6 did not, suggesting that Sgs1 does not suppresses homeologous recombination through a function in the mismatch repair pathway, but plays a MMR-independent role in suppressing translocations between these divergent sequences [128].

**Figure 5:**
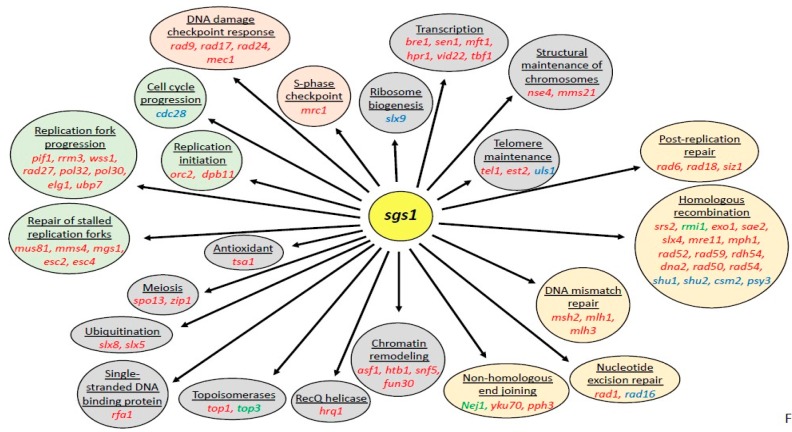
**Genetic interactions of *SGS1*.** Negative genetic interactions of the *SGS1* deletion mutation *sgs1Δ* are shown in red. Mutants that are rescued by *sgs1Δ* are shown in green, and mutations that rescue the *sgs1Δ* mutant are shown in blue. Orange, red, green and gray circles group genes involved in DNA repair, DNA damage and replication stress checkpoints, replication, and ‘other’ functions, respectively. Genetic interactions are primarily based on growth rates, hypersensitivity to DNA-damaging agents, DNA resection rates, mutation frequencies and checkpoint activation.

Deletion of *SGS1* is lethal in combination with numerous mutations (Figure 5), most commonly those that affect recombinational DNA repair, such as *SRS2*, *MUS81/MMS4*, *SAE2*, *MRE11*/*RAD50*/*XRS2* and *RRM3* [49,116,120,129,130,131,132]. The synthetic lethality of the *sgs1Δ sae2Δ* mutant further underscores the role of Sgs1 in DSB end resection; its suppression by *YKU70* deletion suggests that the DSB end-binding protein Ku70 blocks DSB end-resection by the exonuclease Exo1 [133]. Inviability of the *sgs1Δ srs2Δ* and *sgs1Δ mus81Δ* mutants and the severe growth defect of the *sgs1Δ rrm3Δ* mutant are suppressed by deleting *RAD51*, suggesting that, similar to Srs2, Rrm3 and Mus81/Mms4 prevent the formation of recombination intermediates that require the Sgs1/Top3/Rmi1 complex for dissolution [130,132,134,135]. Similarly, combining the *rqh1Δ* mutation with *mre11Δ* or *mus81Δ* mutations is synthetically lethal in *S. pombe* [121,136]. Table 3 provides a comparison of the cellular functions of Sgs1 and Rqh1 indicated by the phenotypes and genetic interactions of their deletion mutants.

### 6.2. Roles of Sgs1 in DSB Repair

**Figure 6:**
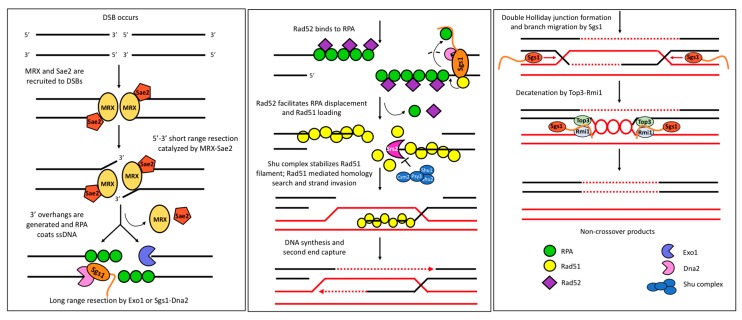
**Early and late roles for Sgs1 in DSB repair by homologous recombination.** In addition to long-range resection and double-Holliday-junction dissolution, Sgs1-Top3-Rmi1 has also been implicated in D-loop reversal. Genetic evidence suggests that the Sgs1-Rad51 interaction promotes homologous recombination; a model has been proposed wherein the acidic region of Sgs1 acts as a DNA mimic that can compete with ssDNA for RPA binding, thereby facilitating initial loading of Rad51 [57]. Shu complex proteins promote Rad51 filament formation by antagonizing the antirecombinase Srs2 and by interacting with Rad51-Rad55-Rad57 [148,149,150].

Sgs1 participates in both early and late steps of homologous recombination (Figure 6). Following formation of a DSB, phosphorylation of Sae2 by cyclin-dependent kinase Cdk1 stimulates DNA end resection at the 5′ end by the Mre11/Rad50/Xrs2 (MRX) nuclease complex, producing short 3′ ssDNA overhangs [126]. This initial processing is crucial since it directs the DNA break to HR and inhibits NHEJ, but it is not sufficient for the formation of the recombinogenic Rad51 filament for error-free repair. In fact, when resection stops at this step (in the *sgs1Δ exo1Δ* mutant), repair in the presence of Rad51 is mutagenic [57]. Thus, the trimmed DSB ends produced by MRX/Sae2 subsequently undergo long-range resection either by the 5′-3′ exonuclease Exo1 or the concerted action of the DNA helicase/nuclease Sgs1/Dna2 [125,126,142,143]. Although Sgs1 and Exo1 are equally capable of fully resecting DSB ends, Sgs1 possesses intrinsic nucleosome repositioning activity, implying that the Sgs1-mediated resection machinery would be less prone to encountering nucleosome impediments than Exo1 [144,145]. Notably, *in vitro*, resection activity of Exo1, but not Sgs1-Dna2, could be stimulated by replacement of H2A with H2A.Z [145]. Together with the finding that H2A.Z is incorporated into chromatin in an Swr1-depedent manner near DSB sites [146,147] and genetic evidence from the *sgs1 swr1* mutant, this suggests that the less stable H2A.Z allows efficient chromatin resection by Exo1 specifically at DSBs [145]. Thus, Exo1 nuclease activity appears to be limited to DSBs and to the replication fork where its activity in mismatch repair is not likely to be impeded by nucleosomes. In contrast, Sgs1 appears to resect efficiently through any nucleosomes and may therefore depend on its numerous protein–protein interactions to target its activity carefully throughout the genome.

Sgs1 can be recruited to dsDNA ends either in its inactive ATP-free state or via the Top3/Rmi1 complex, which promotes ATP-dependent recruitment of Sgs1 to DNA ends; however, Sgs1 fails to initiate translocation in both scenarios [144]. Only addition of Dna2 activates long-range translocation activity of Sgs1, both independently as well as in cooperation with Top3-Rmi1. An *in vitro* study identified Sgs1, Dna2 and RPA as the minimal resection complex where Sgs1, stimulated by RPA, unwinds DNA, producing the substrate for Dna2 to degrade [50]. Another *in vitro* study showed that RPA, in the presence of ATP, only stimulates short-range helicase activity of Sgs1 [144], suggesting the need for additional factors.

The resected 3′ ssDNA tail is immediately bound by RPA, which subsequently binds Rad52 [151]. Binding of Rad52 to RPA facilitates RPA displacement by Rad51, which assembles as a nucleoprotein filament and facilitates strand invasion into a homologous duplex [151]. The binding site of Rad51 maps to a region immediately downstream of the RQC domain of Sgs1 and the sgs1-F1192D (FD) mutant fails to bind Rad51 [57]. Unlike the *SGS1* deletion, the *sgs1-FD* allele does not affect the viability of the *srs2Δ* mutant and suppresses its hypersensitivity to HU and MMS, suggesting that *sgs1-FD* is a hypo-rec allele of *SGS1*. Additional positive and negative genetic interactions of the *sgs1-FD* allele that are also distinct from those of the *SGS1* deletion suggest that the Sgs1/Rad51 interaction promotes homologous recombination [57]. On the other hand, a recent *in vitro* study found that similar to Srs2, Sgs1 can dismantle Rad51 filaments, albeit with some mechanistic differences. Sgs1 was able to translocate on RPA-coated ssDNA without dislodging RPA, but stripped off Rad51 [152]. In contrast to Srs2, which strips off RPA and Rad51 by stimulating Rad51 ATP hydrolysis activity, there was no difference in the ability of Sgs1 to dislodge ATPase-deficient or ATPase-proficient Rad51 from ssDNA [152,153]. This Sgs1 activity may be used to prevent the formation of deleterious HR intermediates at stalled replication forks, to disrupt heteroduplexes, or to displace the invading Rad51 filament in D-loops, thereby channeling HR intermediates into the synthesis-dependent strand annealing (SDSA) pathway of DSB repair that produces noncrossover products [152,154,155,156].

If the second end of the processed DSB is also captured to form a double Holliday junction, Sgs1, in a complex with Top3/Rmi1, branch migrates the Holliday junctions to form a hemicatenane structure that is dissolved by the strand passage activity of Top3 [156]. Stimulated by Rmi1, this step produces exclusively non-crossover products [156,157]. In the absence of Sgs1/Top3/Rmi1 complex activity, double Holliday junctions are resolved by endonucleases, which generates both crossovers and noncrossovers.

Recent evidence suggests that the role of Sgs1/Top3/Rmi1 in HR is regulated by the structural maintenance of chromosomes (SMC) complex Smc5/6, which is known to function in DNA repair and replication and whose functions are mediated by the interacting SUMO ligase Mms21 [158]. Sgs1 physically interacts with Smc5/6 and it has been proposed that Smc5/6 binding to DNA damage sites causes its SUMOylation via Mms21, followed by Sgs1 recruitment to Smc5/6 through its SUMO interacting motifs and later Smc5/6-dependent SUMOylation of Sgs1 and Top3 to activate recombination [53,159]. Indeed, disrupting SUMOylation or Sgs1 interaction with Smc5/6 elevates joint molecule accumulation, supporting a role for the Sgs1-Smc5/6 interaction in HR, and reduces Sgs1/Top3/Rmi1 foci formation in the presence of DNA damage [106].

Although mechanistically still unclear, Rqh1 has also been proposed to be involved in early and late steps of HR. Partial suppression of HU and UV sensitivities of *rqh1Δ* cells by expression of *E. coli* Holliday junction resolvase RusA implicate Rhq1 in preventing Holliday junction accumulation [160]. However, in vitro studies showing that Rqh1 can process Holliday junctions like Sgs1 and BLM remain to be performed. Loss of *rhp55/rhp57* (Rad55/57 homologs) was able to suppress the HU sensitivity of *rqh1Δ* mutants, which was dependent on *rhp51*, suggesting a role of Rqh1 downstream of Rhp55/57 [161]. In support of an early role of Rqh1 in HR, it was shown that Rqh1 foci formed earlier than Rhp51 (Rad51 homolog) foci upon exposure to UV [141]. However, *S. cerevisiae* and *S. pombe* differ in the contribution of the RecQ helicase to DSB resection. While budding yeast Exo1 and Sgs1 can independently perform sufficient long-range resection, Exo1 is responsible for the majority of extended resection in *S. pombe* and Rqh1-Dna2 makes only a minor contribution to overall DSB resection [162]. The strong resection defect observed in *exo1Δ* mutants is partially rescued by loss of Pxd1, which inhibits the nuclease activity of Dna2 [163]. Pxd1 was identified as a regulator of DSB repair by the single-strand annealing (SSA) pathway; it physically interacts not only with Dna2, but also with Rad16, thereby regulating the activity of the two nucleases and improving SSA outcomes [163]. On the other hand, Exo1 and Rqh1-Dna2 play redundant roles in resection of uncapped telomeres arising from deletion of *pot1* [164].

### 6.3. Roles of Sgs1 at the Replication Fork

Although the molecular mechanisms of Sgs1 function at replication forks are still unclear, several studies have linked Sgs1 to the replisome, suggesting roles in sensing DNA damage and overcoming barriers to replication fork progression. The peak of Sgs1 levels in S-phase, colocalization of Sgs1 with the Orc2 subunit of the origin recognition complex, and physical interaction with replisome components, such as Top1, Top2, RPA and DNA polymerase ε support placement of Sgs1 at unperturbed replication forks [19,20,58,165,166]. Chromatin-immunoprecipitation and immunofluorescence microscopy have also placed Sgs1 at sites of *de novo* DNA synthesis, although it remains to be clarified if Sgs1 directly influences normal DNA replication or if Sgs1 tags along so it is present in the event of fork stalling or collapse [166]. Better established is Sgs1′s importance for the response to replication stress. Sgs1 acts in the intra S-phase checkpoint, which stabilizes the replisome in the event of stalling and halts cell cycle progression until DNA is repaired [16,165,167]. Sgs1 acts upstream of the DNA damage checkpoint kinase Rad53 and colocalizes with it in S-phase specific foci [165]. Sgs1 may also help to prevent DNA breaks at stalled forks by using its ATPase/helicase activity to reverse chicken-foot structures that can form if progression of the replisome is blocked by a DNA lesion on the leading strand [168]. The branch migration activity of Sgs1 could help convert regressed forks into normal fork structures, thereby facilitating replication restart.

*sgs1* mutants display faster progression through S-phase, suggesting defective checkpoint signaling, but tend to accumulate in G2/M phase, likely resulting from unrepaired S-phase DNA damage [165,169]. A similar effect is observed in MMS-treated *rqh1Δ* cells [170] and even though these cells arrest DNA replication in response to HU normally, they exhibit defective chromosome segregation [21]. RusA, an *E. coli* resolvase, is able to suppress the hypersensitivity of *rqh1Δ* mutants to fork-stalling agents such as MMS and HU, but not to CPT and γ-rays that cause fork collapse, suggesting different roles for Rqh1 in rescue of stalled and collapsed forks [139,160]. That cells expressing helicase-defective Sgs1 are significantly less sensitive to CPT than *sgs1Δ* cells suggests that the role of Sgs1 in the repair of collapsed forks does not depend on its helicase activity but may involve recruitment of Top3 or other repair factors. Similarly, the role of Rqh1 in the repair of collapsed forks may involve activation of DNA repair factors, such as Top3, which can modulate DNA topology for recombinational repair [139,171,172].

Holliday junction formation has been demonstrated at stalled forks in checkpoint-deficient cells [173] and there is evidence that Sgs1-Top3 activity is involved in resolving these structures. The X-shaped structures at damaged replication forks in cells lacking Sgs1 or Top3 are also displayed by *ubc9* and *mms21* mutants [55]). Similar amounts of X-shaped structures in the double mutants indicate that Ubc9/Mms21 cooperates with Sgs1/Top3 to resolve these structures during replication, possibly via Ubc9-mediated SUMOylation of Sgs1 [55,74]. A functional overlap in resolving stalled replication forks has also been observed for Sgs1-Top3 and the structure-specific endonuclease Mus81-Mms4 [131].

In DNA-damage checkpoint activation, Sgs1 works parallel to Rad9, an adaptor for activation of the central effector kinase Rad53 in response to DNA-damage [174]. Genetic studies of intra-S-phase checkpoint activation show that Sgs1 acts in parallel to Rad24, which loads the PCNA-like Mec3/Rad17/Ddc1 DNA-damage sensor onto ssDNA, and is required for full Rad53 activation [58,165]. Further, Sgs1 functionally interacts with Dia2 and Mph1 in the restart of replication forks stalled at MMS-induced DNA lesions [98]. In HR, the Sgs1 and Mph1 helicases act through separate mechanisms in the suppression of crossover products [157,175,176,177,178]. Similarly, during replication, Sgs1/Top3/Rmi1 is thought to dissolve recombination intermediates that arise from sister-chromatid recombination at broken replication forks [49,179]. Sgs1 also acts on damaged replication intermediates to provide substrates for fork rescue mechanisms, such as Mph1- and Mus81/Mms4-mediated pathways [180]. The physical interaction of Sgs1 with Dia2, an F-box protein component of the SCF E3 ubiquitin ligase complex, appears to recruit Sgs1 to stalled forks by mediating degradation of the fork protection complex component Mrc1 and is needed for Rad53 activation in response to replication stress [98]. These insights into roles of Sgs1 at damaged forks, although still limited, are beginning to provide some mechanistic explanations for the impaired recovery of the *sgs1Δ* mutant from prolonged fork arrest [166].

Synthetic lethality between *sgs1Δ* and *slx4Δ* mutations suggests that the Slx1-Slx4 complex functions redundantly with Sgs1/Top3/Rmi1 on stalled forks, cleaving and decatenating them, respectively [181,182]. In addition to its role in dissolving recombination intermediates during replication stress, there is evidence that Sgs1 contributes to the stabilization of polymerases α/primase and ε at stalled forks [58,166]. *pol2* mutants are defective in the activation of the intra S-phase checkpoint, and this defect is epistatic with *sgs1*, suggesting that Sgs1 and DNA pol ε function in the same pathway, in parallel to Rad24/Mec3/Rad17/Ddc1, to activate Rad53 in presence of HU [165]. Deletion of *SGS1* in a checkpoint deficient *mec1-100* mutant results in complete dissociation of replicative DNA polymerases, impaired recovery from replication fork arrest and a synergistic increase in the GCR rate, providing evidence that Sgs1 and Mec1 independently contribute to genome stability [183]. The suppression by *mms2* and *ubc13* mutations of the severe growth defects of *sgs1Δ* mutants lacking the DNA polymerase subunit Pol32 suggests a role for Sgs1 in the error-free Rad6 DNA damage tolerance (DDT) pathway [184,185]. However, requirement of Sgs1 for efficient PCNA monoubiquitination during replication stress also suggests a role for Sgs1 in the error-prone translesion DNA synthesis (TLS) pathway [186]. Similar to *sgs1Δ*, *rqh1* mutations reduce the growth rate of *S. pombe* lacking DNA polymerase subunits such as those of DNA pol δ and ε [23].

## 7. Meiosis

Due to its role in homologous recombination Sgs1 was also expected to be required for successful completion of meiosis. Indeed, deletion of *SGS1* reduces meiotic product (tetrad) formation and spore viability; however, unlike in mitotic cells, meiotic crossover formation is not markedly increased [20,187,188,189,190,191], suggesting differences in the roles of Sgs1 in mitotic and meiotic recombination. Notably, the helicase activity was found to be dispensable for Sgs1′s meiotic function, suggesting that Sgs1 serves structural or regulatory functions in interaction with other meiotic factors, such as recruitment of Top3 and Top2 or other factors that interact with the meiosis-essential domain of Sgs1 that spans residues 126–595 [188,189,192]. Sgs1 also colocalizes with Zip3, a component of the synapsis initiation complex, and absence of Sgs1 correlates with an increase in synapsis initiation complexes [187]. Since Zip3 interacts with HR proteins such as Rad51 and Mre11, and its absence delays synaptonemal complex formation [193], Sgs1 may help regulate Zip3 activity. Indeed, longer persistence of fully synapsed chromosomes, suggesting failure to timely exit the meiotic pachytene stage, is thought to be responsible for poor spore formation in the *sgs1Δ* mutant [187].

Moreover, *sgs1Δ* mutants exhibit elevated meiotic chromosome missegregation and most of these events are associated with chromosome nondisjunction rather than defects in sister chromatid separation [20]. *SGS1*/*rqh1* deletion is synthetically lethal with *mus81Δ* and *mms4Δ*, raising the possibility of overlapping roles of Sgs1 and the structure-specific endonuclease Mus81-Mms4 in meiotic recombination [139,182]. Lethality was shown to result from unresolved joint molecules that formed during meiotic recombination, suggesting that Sgs1/Rqh1 and Mus81/Mms4 collaborate in mediating efficient joint molecule resolution to promote proper segregation of homologs [194,195,196,197].

The ZMM proteins (Zip1/Zip2/Zip3/Zip4, Msh4/Msh5 and Mer3) function in synaptonemal complex assembly and recombination [198]. Synapsis and crossover formation are impaired in *zmm* mutants, but are restored by deletion of *SGS1* [190]. This suggests that by suppressing the anti-crossover activity of Sgs1, ZMM proteins, also known as the synapsis initiation complex (SIC), promote meiotic chromosome synapsis and crossovers [190]. Rqh1, on the other hand, promotes meiotic recombination in *S. pombe*, which lacks ZMM proteins and crossover interference. Rqh1 may act on fission-yeast-specific meiotic recombination intermediates, such as single Holliday junctions, rather than the double Holliday junctions predominantly found in budding yeast meiosis [197,199,200].

## 8. Possible Functions in Excision Repair

Nucleotide excision repair (NER) primarily repairs DNA lesions that distort the DNA helix, such as UV-induced pyrimidine dimers or bulky DNA adducts, whereas base excision repair (BER) removes bases damaged primarily by oxidation, alkylation or deamination [201,202,203]. Although human NER and BER proteins interact with RecQ helicases, including BLM, no such evidence exists in yeast [204,205,206,207,208]. *sgs1* mutants are only marginally sensitive to UV radiation, but one study suggests that Sgs1 and the NER protein Rad16 may work in a common pathway to repair UV-induced damage [92]. The authors showed that a *RAD16* deletion partially rescues the hypersensitivity of the *sgs1Δ* mutant to MMS and H_2_O_2_, suggesting that Rad16 might direct formation of a substrate that requires dissolution by Sgs1.

A contribution of Sgs1 to BER is less likely. Sgs1 functionally interacts with the 5′ flap-endonuclease Rad27, which besides its well-understood function in Okazaki fragment maturation also plays a role in long-patch BER [202,209]. Sgs1 also functionally interacts with the ROS-scavenging enzyme Tsa1, which suppresses genome rearrangements and is required for normal growth in the absence of Sgs1 [210]. Studies are needed to better delineate possible contributions of Sgs1 to excision repair.

## 9. Telomere Length Maintenance

Several studies suggest a role for RecQ helicases in telomere maintenance, most prominently a role of human WRN whose absence causes telomere defects and rapid, adult-onset aging in Werner syndrome [211,212,213]. Deletion of *SGS1* does not lead to telomere length changes [214]. However, its exquisite ability to unwind G-G paired DNA, such as G-quadruplexes, in vitro extends to telomeric sequences and may be important *in vivo* for ensuring faithful chromosome segregation [80]. Sgs1 also contributes to telomeric end processing, where it might be needed for unwinding, especially when ends form G4 structures, to provide substrates for exonucleases to produce the 3′ G-strand overhangs [214]. Sgs1 has also been proposed to function in telomere lengthening via the alternative lengthening of telomeres (ALT) pathway, which relies on HR and is independent of telomerase [215,216]. Deletion of *SGS1* in telomerase mutants *est2Δ* and *tlc1Δ* precludes formation of Type II survivors, which amplify TG_1-3_ -telomeric repeats [217,218,219]. It is thought that Sgs1 performs Holliday junction dissolution or facilitates replication of T-circles that are involved in elongation of telomeres via rolling-circle replication [140,219,220]. Consistently, *sgs1Δ tlc1Δ* and *sgs1Δ est2Δ* mutants display rapid telomere shortening compared to the single telomerase mutants, implying that Sgs1 reduces the rate of telomere shortening by homologous recombination [217,218]. Furthermore, *sgs1Δ tlc1Δ* mutants display reduced recombination frequency and accumulate X-shaped structures at telomeres in a Rad52-dependent manner, indicating a role of Sgs1 in resolving these recombination intermediates and, thus, preventing premature senescence of *tlc1Δ* mutants [221]. Whereas Sgs1 resolves recombination intermediates and prevents premature senescence of *tlc1* mutants, the Mph1 helicase promotes premature senescence in *tlc1* mutants by causing telomere uncapping and recombinogenic ssDNA accumulation [222], which may form recombination intermediates that would require Sgs1-Top3-Rmi1 for resolution. Notably, in human cells, a physical interaction between BLM-TOP3A-RMI and FANCM, the human Mph1 homolog, acts to restrict the ALT pathway [223]. It will be interesting to determine if a similar physical interaction also exists between Mph1 and Sgs1, and if it impacts telomere length maintenance in yeast. Finally, SUMOylation of Sgs1 at lysine 621 is required for its role in the survival of telomerase-deficient cells by ALT [70,73,74]. Similarly, *S. pombe* Rqh1 is SUMOylated and protects telomeres from breaking, loss and entanglement in cells lacking the telomeric maintenance protein Taz1 [75]. The protective role of Rqh1 at telomeres is also supported by the synthetic lethality of the *pot1Δ* and *rqh1Δ* mutations [224].

## 10. Aging and Transcription

The average and maximum life-span of yeast cells lacking Sgs1 helicase activity is reduced by nearly half, suggesting a significant role of Sgs1 in preventing premature cellular aging [18,171]. Increased genomic instability due to recombination defects in the absence of Sgs1 is one of the major causes of reduced lifespan, but there may be additional contributions from other causes [225]. For example, extrachromosomal rDNA circles (ERCs), which were proposed to cause premature aging in yeast, accumulate at an increased rate in *sgs1* mutants [226]. However, another study found no significant difference in ERC accumulation between *sgs1Δ* and wildtype cells, calling into question the association between ERCs and reduced lifespan of *sgs1Δ* mutants [227]. Increased oxidative stress in *sgs1* mutants and mitochondrial dysfunction due to mtDNA mutations could be other potential causes of *sgs1Δ* mutant’s aging [209,228]. Indeed, human RecQ4 has been shown to localize to mitochondria where it preserves mitochondrial DNA integrity; however, it remains to be determined if Sgs1 contributes to mitochondrial function in yeast [229].

Nucleolar defects arising from the absence of Sgs1 may also contribute to the shortened lifespan of the *sgs1Δ* mutant. Sir3, which silences transcription at telomeres, rDNA and mating loci, localizes to the nucleolus of *sgs1Δ* cells after only nine generations [18,230]. Sgs1 localizes to the nucleolus and *sgs1Δ* cells display nucleolar enlargement and premature nucleolar fragmentation [18]. Nucleolar localization implicates Sgs1 in rRNA synthesis where it may be involved in unwinding of G4-DNA, a common feature of rDNA [80]. Recently, Sgs1 was shown to physically interact with Rio1 to recruit it to rDNA, where Rio1 is involved in rDNA replication [101,102]. Interaction of Sgs1 with the transcription factor Gis1 raises the possibility of additional contributions of Sgs1 to transcription [97].

## 11. Other RecQ-Like DNA Helicases in Yeast

Both budding and fission yeast were believed to possess a single RecQ helicase, Sgs1 and Rqh1, respectively. However, bioinformatics analysis identified another putative yeast RecQ helicase, Hrq1, based on its similarity to human RecQL4 [231]. In contrast to Sgs1, the 3′-5′ helicase activity of Hrq1 is restricted to unwinding duplex DNA with 3′ overhangs. Hrq1 is also stimulated by a fork structure and possesses DNA strand annealing activity [232]. Deletion of *SGS1* in an *hrq1Δ* mutant has additive effects on the mitotic recombination rate, accumulation of spontaneous mutations and growth, suggesting that Hrq1 functions independently of Sgs1 in DNA recombination and repair [233]. Deletion of fission yeast Hrq1 leads to genome instability and hypersensitivity to DNA damaging agents [234]. Like Sgs1, Hrq1 is important for telomerase-independent telomere maintenance [235]. The epistatic relationship between *HRQ1* and *RAD4* and the physical interaction between the two proteins in a yeast-two-hybrid assay suggest that Hrq1 may also have an NER-related function [236]. Proteomic screens have identified two ubiquitination (K366 and K872) and one phosphorylation (S17) site in Hrq1, but their functional significance is unknown [72,237]. Future studies aimed at identifying additional genetic interactions and Hrq1-binding partners will help to clarify the roles of the second yeast RecQ helicase family member in DNA metabolism.

Sequence homology searches revealed two additional RecQ-like proteins in fission yeast, Tlh1 and Tlh2, that show significant sequence homology with other known RecQ helicases between residues 1180 to 1820 [238]. They are telomere-linked DNA helicases that contribute to telomere maintenance in the absence of telomerase. Similar to budding yeast Sgs1, deletion of *Candida albicans* Sgs1 causes hypersensitivity to DNA-damaging agents [239]. In contrast, *Candida glabrata sgs1Δ* mutants display wildtype growth in the presence of DNA-damage-inducing agents, suggesting that CgSgs1 may not be involved in DSB repair [240].

## 12. Outlook

Data accumulated over the past 25 years decidedly point to a role for Sgs1 in maintaining genome stability via its roles in DNA recombination and at the replication fork. However, important questions remain to be answered. For example, does the physical interaction of Sgs1 with constitutive components of the replisome [165] imply a function of Sgs1 during unperturbed DNA replication or does Sgs1 simply move along with the replisome to be available if the fork is damaged? How is the interaction of Sgs1 with over a dozen proteins that have functions in several different DNA metabolic pathways regulated? Only a few of these interactions are understood at the molecular level (Top3-Rmi1, RPA, Rad51, Rad53) and a model has been proposed wherein the coordinated interaction of Sgs1 with Rad51 and RPA promotes homologous recombination [57]; however, binding sites for the vast majority of interacting proteins have only been narrowed down to several hundred residues, which has limited our insight into the functional significance of most Sgs1 interactions. Other questions include: How does Sgs1 suppress non-allelic, interchromosomal recombination events [122,241,242]? Does the increased presence of reactive oxygen species in the *sgs1Δ* mutant [209] point to a role of Sgs1 in maintaining mitochondrial health similar to human RecQL4? What is the helicase-independent role of Sgs1 in meiosis [188,189,192]? Here, yeasts provide unique genetic tools to elucidate fundamental mechanisms of meiotic recombination that may eventually help to better understand the cause of subfertility/infertility in Bloom syndrome. In addition to Sgs1 function, questions regarding the structure of Sgs1 also remain. The N-terminal tail of Sgs1 is one of the longest intrinsically disordered regions in the yeast proteome [87]. Does it simply serve as one of the largest protein binding hubs in yeast or does it have additional biochemical and regulatory functions or engage in intramolecular interactions? Is the ability of Sgs1 to unwind G-quadruplexes based on a similar G-specific pocket that allows *E. coli* RecQ to unfold G-G paired DNA [243]? Finding answers to these questions and many others that remain about RecQ helicases in yeast will shed light on fundamental mechanisms of eukaryotic DNA metabolism and continue to provide direction to investigations of human RecQ helicases, at least three of which are associated with incurable cancer predisposition syndromes.

## Figures and Tables

**Table 1:** Physical interactions of Sgs1.

Binding Partner	Name Description	Assay	Protein Function	Sgs1-Interacting Domain	References
Bud27	Bud site selection	Two-hybrid	TOR-dependent gene expression	421–791	[97]
Dia2 ^a^	Digs Into Agar	Affinity capture-western	Component of SCF E3 ubiquitin ligase complex	Full length	[98]
Dna2	DNA synthesis defective	CoIP	DNA-dependent ATPase, helicase & nuclease	Full length	[51]
Gis1	Glg1-2 Suppressor	Y2H	Histone demethylase & transcription factor	420–791	[98]
Mlh1	MutL Homolog	Y2H	DNA mismatch repair	784–1447	[95]
Mlh3	MutL Homolog	CoIP	DNA mismatch repair	Full length	[53]
Mre11	Meiotic REcombination	CoIP, Y2H	DSBR; nuclease subunit of MRX	Full length	[50]
Prp45	Pre-mRNA processing	Two-hybrid	Pre-mRNA splicing	503–739	[99]
Rad16	RADiation sensitive	Y2H	Nucleotide excision repair	421–792	[93]
Rad51	RADiation sensitive	Y2H, pull-down	DSBR; strand exchange protein	1187–1318	[58,91]
Rad53	RADiation sensitive	ITC	DNA damage response kinase	446–456	[60]
Rpa70/Rfa1	Replication Factor A	Y2H, affinity capture-western, reconstituted complex	Subunit of heterotrimeric RPA (ssDNA binding protein)	421–792	[58,100]
Rio1	RIght Open reading frame	Y2H, CoIP	Serine kinase involved in cell cycle regulation and rDNA integrity	Full length	[101,102]
Rmi1	RecQ Mediated genome Instability	CoIP	DSBR; subunit of Sgs1-Top3-Rmi1 complex	1–100	[87]
Rtt107/Esc4	Regulator of Ty1 Transposition	Y2H	DNA repair during S phase; recruits Smc5/6 to DSBs	Full length	[103]
Smt3	Suppressor of Mif Two	Y2H	Ubiquitin like protein	Full length	[104,105,106]
Srs2	Suppressor of Rad Six		DNA helicase; antirecombinase	422–722	[92]
Stu2	Suppressor of TUbulin	Y2H	Microtubule associated protein	Full length	[107]
Top2	TOPoisomerase	Y2H	Relaxes both positively & negatively supercoiled DNA	466–746	[20,86,94]
Top3	TOPoisomerase	Y2H	Relaxes negatively supercoiled DNA, subunit of Sgs1-Top3-Rmi1 complex	1–282	[19,71,85,86]
Ubc9	UBiquitin-Conjugating	Y2H	SUMO-conjugating enzyme	Full length	[56]

^a^ interaction observed during HU/MMS exposure.

**Table 2:** Physical interactions of Rqh1.

Binding Partner	Name Description	Assay	Protein Description	Rqh1-Interacting Domain	Reference
CAF-1 ^a^ (subunit Pcf1)	Chromatin assembly factor	CoIP	Histone chaperone promotes chromatin assembly during DNA repair and replication	Full length	[108]
Top3	Topoisomerase III	CoIP	Relaxes negatively supercoiled DNA	1–322	[78]
RPA (Rad11)	Replication protein A	CoIP	Binds to ssDNA	Full length	[109]
Cdc23	MCM associated protein Mcm10	Two-hybrid	Efficient phosphorylation of MCM complex and pre-RC activation	Full length	[110]
Nse2 ^b^	Non-SMC element SUMO ligase	Biochemical activity	Component of Smc5-6 required for DNA damage response	Full length	[111]
Cbh1	CENP-B homolog	Two-hybrid	Promotes Swi6 association with centromere causing increased silencing	Full length	[110]
Rcl1	rRNA processing protein	Two-hybrid	Nuclease for 18S rRNA production	Full length	[110]
Rmi1	RecQ mediated genome instability protein	Two-hybrid	Holliday junction dissolution	Full length	[110]
Usp104	U1 snRNP-associated protein	Two-hybrid	Splicing factor	Full length	[110]
Pfh1	PiF1 Helicase homolog	Affinity capture-MS	5′-3′ DNA helicase promotes fork progression	Full length	[112]
Spo7	Sporulation protein	Two-hybrid	meiotic spindle pole body component	Full length	[110]
Atg11	Autophagy associated protein	Two-hybrid	Scaffold protein in mitophagy	Full length	[110]
Pli1 ^b^	SUMO E3 ligase	Biochemical activity	Major SUMO ligase, role in telomere maintenance	Full length	[111]

^a^ protects recombination intermediates from disassembly by Rqh1 ^b^ can both SUMOylate Rqh1.

**Table 3:** Comparison of budding yeast Sgs1 and fission yeast Rqh1 properties.

Property ^a^	Sgs1	Rqh1
ATPase/Helicase activity	Yes	Yes
PTMs ^b^	Phosphorylation, SUMOylation	Phosphorylation, SUMOylation
Nucleolar localization	Yes	Yes
Localization in nuclear foci	Yes	Yes ^c^(NLS:^1294^YSRKRKYSTS^1303^)
Homologous Recombination	Yes	Yes
Suppression of homeologous recombination	Yes	n.d. ^d^
Suppression of GCRs ^e^	Yes	Yes (only between gene duplications)
Maintenance of normal lifespan	Yes	Yes
Suppression of meiotic defects	Yes	Yes
Meiosis	Yes	Yes
Meiotic recombination	Yes	No
Activation of intra-S phase checkpoint ^f^	Yes	Yes
Stabilization of replicative polymerases at stalled forks	Yes	n.d.
Telomere maintenance ^g^	Yes	Yes
rDNA maintenance	Yes	Yes
Resistance to DNA damaging agents	MMS, HU, CPT ^h^, IR, H_2_O_2_, Cisplatin, MMC ^i^	MMS, HU, CPT, MMC, IR, UV
Substrates	ssDNA, dsDNA, four-way junctions, forked and G4 DNA	dsDNA

^a^ Functions and properties of Sgs1 and Rqh1 are from references [2,120,137,138,139,140,141] ^b^ post-translational modifications ^c^ predicted using cNLS Mapper ^d^ n.d., not determined ^e^ GCR, gross chromosomal rearrangement ^f^ observed in presence of DNA damage ^g^ in the absence of telomerase activity ^h^ CPT, camptothecin, topoisomerase I inhibitor ^i^ MMC, mitomycin C, DNA inter-strand crosslinking agent.
